# In Memorandum of World Kidney Day: Chronic Kidney Disease: A Common But Often Unnoticed Major Health Problem

**Published:** 2011-03-01

**Authors:** M K Fallahzadeh, M M Sagheb, M H Fallahzadeh

**Affiliations:** 1 Shiraz Health Policy Research Center, Shiraz University of Medical Sciences, Shiraz, Iran; 2 Department of Nephrology, Shiraz Nephro-Urology Research Center, Shiraz University of Medical Sciences, Shiraz, Iran; 3 Department of Pediatric Nephrology, Shiraz University of Medical Sciences, Shiraz, Iran

In the past two decades, the international health efforts have been focused on the control of infectious diseases in developing countries with noncommunicable diseases (NCDs) given a lower priority.[1] However, the incidence of NCDs including chronic kidney disease (CKD) is increasing in developing countries where primary care intervention for CKD is often inadequate.[2] The main international focus is now on NCDs including diabetes mellitus (DM), hypertension, obesity and chronic airway obstruction. Most of the NCDs share common risk factors which are potentially amenable to behavioral modifications including smoking, unhealthy diet, physical inactivity and stress. These diseases can usually be detected by simple tests which are available in primary care settings even in low and middle income countries. CKD which is often caused by underlying diseases such as DM and hypertension can be effectively prevented by detection and treatment of these diseases.[2] According to Kidney Disease Outcomes Quality Initiative (KDOQI) guidelines, CKD is defined as i) Persistent glomerular filtration rate (GFR) < 60 mL/min/1.73 m(2) for ≥ 3 months with or without kidney damage or ii) Kidney damage for ≥ 3 months based on structural and functional anomalies of the kidney with or without decreased GFR.[3]

CKD is now a common health problem globally; up to 10% of world’s population is estimated to suffer from CKD.[4] Number of the patients with end stage renal disease (ESRD) keeps increasing and is now projected to affect more than 2 million people worldwide. Increasing incidence of CKD not only adds burden to global health care resources but also has significant impact on patients and their families. Therefore, it is of great importance to detect and prevent CKD at its early stages.[5]

In 2004, over 700,000 patients were estimated to have CKD in Iran with incidence rate of 173.5/100,000. CKD was responsible for 1,145,654 disability adjusted life years (DALYs). Among the underlying causes for stages of 1 to 4 of CKD in Iranian population, DM and hypertension were the most common ones. In 2008, more than 24,000 patients were estimated to suffer from ESRD (stage 5 of CKD) in Iran. Patients with ESRD constitute the tip of iceberg of patients with varying degrees of CKD.[6] In Iran, the annual number of patients with ESRD beginning maintenance therapy increased by 130% between 2000 and 2006. The incidence of ESRD secondary to DM increased 2 folds from 1997 to 2006 (from 16% to 31%).[7]

Patients with CKD are 5 to 11 times more likely to die prematurely than to progress to ESRD.[8] The high disease burden of CKD, the high cost of treatment and the fact that preventive measures are not yet fully in place in many countries, quantify CKD as a public health problem.[9]

The main risk factors for CKD are DM, hypertension, obesity, aging of the population, dyslipidemia, family history of kidney disease and smoking.[10] Among these risk factors, DM is the leading cause of CKD globally; presently, diabetic nephropathy is estimated to affect 285 million adults worldwide. It is expected to rise by 54% to 839 million by 2030 according to the International Diabetes Federation.[11] Increased prevalence of hypertension from 972 million to 1.5 billion by 2015 is also expected.[12]

The screening and preventive strategies must be well suited to the particular population by considering the availability of human and material resources and require different approaches depending on the race, habitual and socioeconomic status.[5][10] By considering the complex interrelationship between CKD and preventable risk factors, a screening program for hypertension, DM, obesity and proteinuria must occur at primary care level and preferentially be incorporated into the millennium development goals. Although targeted screening has been advocated in high income countries in view of its cost-effectiveness, screening all of the population may be more suitable for less sophisticated health systems; because this will increase detection rate and proper management of other NCDs as well as CKD.[13] Failure to detect CKD at its early stages can result in high morbidity and mortality; however, early detection of CKD is difficult because of its asymptomatic nature.[14]

Dipstick urine examination at least for high risk groups and shared care program are cost-effective for CKD screening and prevention.[5] The cost-effectiveness and improved detection of NCDs has been proven in the Chennai Project in Southern India.[10] Screening for renal diseases by measuring blood pressure and using a single urinary dipstick test in Iranian school-age children was also demonstrated to be cost-effective.[15] Prevention of urinary tract infection as the leading cause of CKD in children with vesicoureteral reflux or obstruction of the urinary tract can be effective in prevention of CKD.

Reducing the rate of hypertension, DM and its attendant cardiovascular disease will involve lifestyle modifications like weight reduction and a healthy diet including decrement in the consumption of saturated fat and synthetic foods and increment in consumption of fruits and vegetables, regular exercise, and cessation of smoking.[10] Decreasing the rate of prescription of nephrotoxic medications also appears to be of great importance.

In patients with CKD, target blood pressure (BP) levels should be < 130/80 mmHg and a tighter BP control (<125/75 mmHg) is recommended in patients with DM or proteinuria > 1 gram/ 24 hours. [10] Angiotensin converting enzyme inhibitors and/or angiotensin receptor blockers are the first choice medications for control of hypertension in patients with CKD and proteinuria. Furthermore, strict glycemic control (HbA1C < 7%) should be the goal in diabetic patients. [10]

Training of nephrologists and sustained education of general physicians and other health care personnel is an essential part of development of preventive measures for CKD. Furthermore, sustained collaboration among governmental agencies, health policy makers, pharmaceutical industry, international societies and philanthropic bodies is necessary. [10]

On March 10, 2011, the sixth World Kidney Day will be celebrated. This annual event has been initiated by International Society of Nephrology and International Federation of Kidney Foundations in order to raise the awareness of health care providers, government health officials and general public about kidney diseases and their attendant complications. World Kidney Day activities will provide a very good opportunity to appreciate and robust the notion that kidney disease is common, preventable and treatable. The theme of this year’s world kidney day is “Protect your kidneys and Save your heart”.[16] CKD is an important and independent risk factor for premature cardiovascular disease, the most important cause of mortality and morbidity worldwide. Screening, early detection and treatment of kidney diseases not only improve the long-term renal but also cardiovascular outcomes. [16]

In summary, CKD and its underlying diseases are becoming a major health problem in developing countries. Screening for the risk factors of CKD and their proper management has a great role in prevention of CKD in children and adults. This demands collaboration between health policy makers and governmental agencies, educated healthcare staff, sufficient financial support and extensive public education.
